# Rapidly Progressive High-Grade Leiomyosarcoma in an Elderly Patient

**DOI:** 10.7759/cureus.67919

**Published:** 2024-08-27

**Authors:** Elias Uyar, Sabrina Carpintieri, Alberto Gomez Veliz

**Affiliations:** 1 Internal Medicine, Ross University School of Medicine, Miami, USA; 2 Medicine, Ross University School of Medicine, Miami, USA; 3 Internal Medicine, Jackson Memorial Hospital, Miami, USA

**Keywords:** extraskeletal osteosarcoma, heart failure with reduced ejection fraction, metastatic lung nodule, acute pulmonary embolism, leiomyosarcoma of the thigh

## Abstract

An 84-year-old female with a history of hypertension, diabetes, and hypothyroidism initially presented in November 2023, with a rapidly enlarging (19.5 cm) left proximal thigh mass. Biopsy diagnosed high-grade leiomyosarcoma, which doubled in size within two weeks, confirming aggressive biology. In January 2024, the patient, who had been ambulating independently one year prior to her diagnosis, underwent radical resection and femoral neurolysis, and initiated radiotherapy, without receiving neoadjuvant chemotherapy due to cachexia.

Three months postoperatively, in April 2024, the patient presented with acute respiratory distress, requiring 4L oxygen, and bilateral lower extremity edema. Imaging revealed numerous bilateral pulmonary metastases and an acute pulmonary embolism in the right inferior segment branch. She was admitted with decompensated heart failure, an ejection fraction of 30-45%, and extensive metastatic leiomyosarcoma. Despite anticoagulation, her status rapidly declined.

This case highlights the challenges of rapidly progressive sarcomas characterized by fulminant growth and early metastatic spread. Earlier treatment with neoadjuvant chemotherapy prior to surgery may have improved outcomes but was precluded by the patient's frailty. After a multidisciplinary discussion, the decision was made to transition to hospice care. This case also underscores the potential for rapid clinical deterioration with metastatic leiomyosarcoma. It highlights the challenges of managing complications from aggressive malignancies, especially in frail patients, where treatment-related toxicities may outweigh the benefits. Careful patient selection for cancer-directed therapies via multidisciplinary input is imperative.

## Introduction

Leiomyosarcoma is a rare and aggressive type of soft tissue sarcoma that originates from smooth muscle cells. It accounts for approximately 10-20% of all soft tissue sarcomas and can occur in various locations throughout the body, including the extremities, retroperitoneum, and uterus [1]. High-grade leiomyosarcomas are particularly challenging to manage due to their rapid growth and high propensity for metastasis.

Genetic alterations in leiomyosarcomas frequently involve mutations in tumor suppressor genes, with RB1 and PTEN being particularly significant, as evidenced by losses in chromosomal regions 10q and 13q, which are commonly observed [2]. The genetic landscape of leiomyosarcoma is characterized by a wide array of cytogenetic and molecular variations. This diversity in genetic abnormalities contributes significantly to the highly heterogeneous nature of the disease, making it challenging to develop targeted therapies and predict clinical outcomes.

In evaluating suspected sarcomas, initial imaging with CT or MRI is crucial to assess tumor extent, relationships with surrounding structures, and potential biopsy targets. CT is particularly useful for retroperitoneal and visceral lesions while MRI excels in examining tumors in the head, neck, and extremities [3]. The choice between CT and MRI often depends on local availability. Following imaging that suggests a sarcoma, an image-guided core needle biopsy is essential for diagnosis, as fine-needle aspiration does not provide sufficient tissue for accurate classification [4,5]. This systematic approach combining advanced imaging and targeted biopsy is fundamental for guiding appropriate treatment strategies for sarcomas.

For patients with operable leiomyosarcomas, surgeons typically perform tumor resection as the first-line treatment. Oncologists often supplement this approach with chemotherapy and radiation therapy as part of a multifaceted treatment plan, tailoring these additional interventions to each patient's specific case [1,6]. The management of such cases often requires a delicate balance between aggressive treatment and quality-of-life considerations. This report aims to discuss the challenges encountered in treating this patient, including the decision to forego neoadjuvant chemotherapy, the complications that arose postoperatively, and the ultimate transition to palliative care. Managing elderly patients with aggressive soft tissue sarcomas presents significant challenges due to their poor prognosis. However, emphasizing multidisciplinary approaches and individualized treatment plans can potentially enhance quality of life.

This case report presents an 84-year-old female with a rapidly progressing high-grade leiomyosarcoma of the left proximal thigh. The case is notable for several reasons: the patient's advanced age, the extremely rapid tumor growth, and the swift clinical deterioration following surgical intervention. It illustrates the complex decision-making process in managing aggressive malignancies in elderly, frail patients and highlights the potential for rapid disease progression and metastasis in high-grade leiomyosarcomas.

## Case presentation

An 84-year-old female with a complex medical history, including hypertension, type 2 diabetes mellitus, hypothyroidism, and a previous pulmonary embolism, presented to the emergency department on 04/19/2024, with complaints of progressive shortness of breath and worsening bilateral lower extremity edema. The patient's vital signs were recorded in Table 1. Initial laboratory tests were performed prior to admission (Table 2). Family history was non-contributory. The patient denies any alcohol, tobacco, or recreational drug use.

**Table 1:** Vital signs recorded in the Emergency Department on 4/19/2024 bpm: beats per minute; BRPM: breaths per minute

Vital Signs	Recorded Value
Temperature	37^o^C (98.6^o^F)
Blood Pressure	142/83 mmHg
Heart Rate	101 bpm
Respiratory Rate	20 brpm
Oxygen Saturation	92% on room air

**Table 2:** Initial laboratory test on 4/19/2024 (H): Indicates a high value outside the reference range; (L): Indicates a low value outside the reference range

Laboratory Test	Laboratory Result	Reference Range
Glucose	137 mg/dL (H)	70-99 mg/dL
Sodium	139 mmol/L	135-145 mmol/L
Potassium	4.4 mmol/L	3.5-5.1 mmol/L
Calcium	10.6 mg/dL (H)	8.6-10.3 mg/dL
Blood Urea Nitrogen	15 mg/dL	7-20 mg/dL
Creatinine	0.80 mg/dL	0.6-1.2 mg/dL
NT-proBNP	681.0 pg/mL (H)	<125 pg/mL
WBC Count	2.9 x 10³/mcL (L)	4.5-11.0 x 10³/mcL
RBC Count	4.33 x 10⁶/mcL	4.2-5.4 x 10⁶/mcL
Neutrophil (%)	76.1 % (H)	40-60%
Lymphocyte (%)	16.1 %	20-40%
Monocyte (%)	5.8 % (L)	2-8%
Eosinophil (%)	1.4 %	1-4%
Absolute Lymphocyte	0.5 x 10³/mcL (L)	1.0-3.0 x 10³/mcL
Absolute Monocyte	0.2 x 10³/mcL	0.2-0.8 x 10³/mcL
Absolute Eosinophil	0.04 x 10³/mcL (L)	0.05-0.5 x 10³/mcL

**Figure 1:**
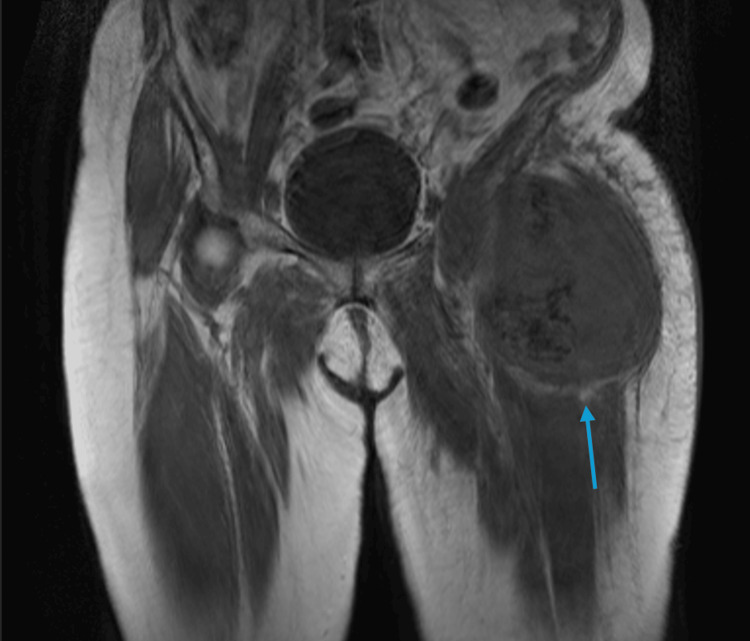
MRI lower extremity without contrast The blue arrow indicates a large heterogenous mass measuring 10 x 12.5 x 13.4 cm (anterior-posterior x transverse x craniocaudal) abutting the anterior inferior iliac spine with internal calcification and peritumoral edema consistent with high-grade soft tissue sarcoma. This MRI was taken on 11/14/2023, and the mass doubled in size in the next two weeks.

**Figure 2:**
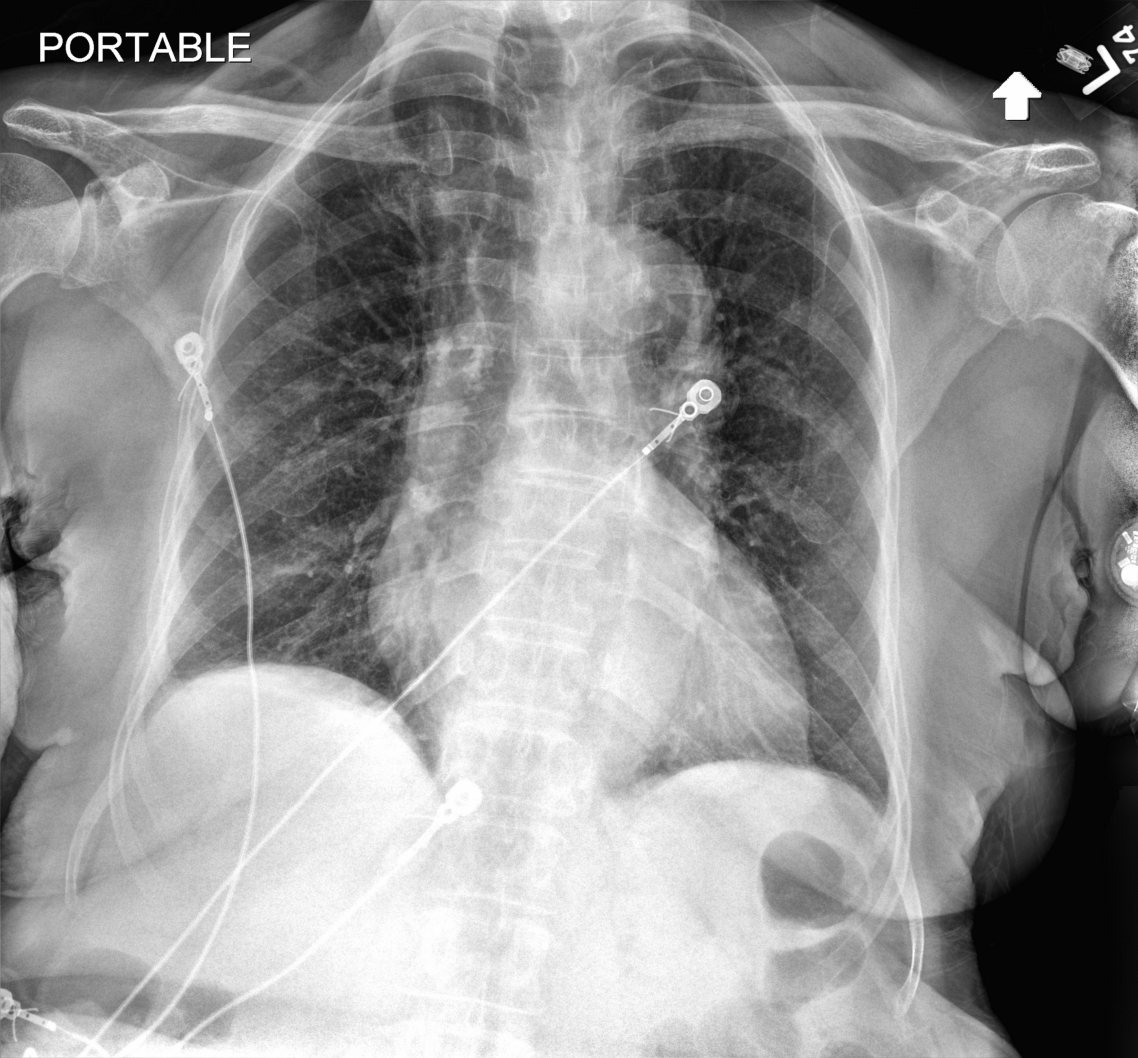
Chest X-ray on 12/08/2023 The cardiac silhouette is within normal limits for technique. The mediastinum is midline. The thoracic aorta is mildly tortuous with calcifications at its arch. The lungs are well aerated showing mild hyperinflation without consolidation. No indication of edema or airspace disease.

The patient's history of leiomyosarcoma began on 11/14/2023 when she presented to the emergency department for a left leg mass. The mass was first noticed three weeks prior, with severe pain since the onset. A left thigh mass needle biopsy confirmed leiomyosarcoma, FNCLCC grade 1, as shown in Figure 1. Notably, within two weeks of the biopsy, the mass had doubled in size and was firm to touch, indicating the aggressive nature of the tumor. On 12/8/2023, the patient was worked up for complaints of dyspnea. A chest X-ray revealed no significant findings (Figure 2), and specifically, no mass or evidence of metastasis was noted.

Following a multidisciplinary discussion, it was determined that the patient was not a candidate for chemotherapy due to her advanced age and comorbidities. The decision was made to proceed directly to surgery, which was performed on 01/09/2024. The procedure involved radical resection and neurolysis of the femoral nerve. The patient was walking independently and fully independent prior to her surgery; after the surgery, the patient uses a cane to ambulate.

The surgical specimen revealed a 19.5 x 18.1 x 13.4 cm soft tissue mass with an overlying 15.6 x 5.3 cm ellipse of skin. Upon sectioning, the tumor was found to be well-circumscribed, partially encapsulated, and solid with a central cystic area measuring 10 x 9 x 5 cm. Areas of calcification and hemorrhage were noted within the tumor.

Postoperatively, the biopsy revealed the patient's tumor grade to be a stage 3b left thigh leiomyosarcoma and began adjuvant radiotherapy. As of 04/18/2024, she had received 12 fractions of a planned 60 Gy in 30 fractions (50 Gy/25 fx + boost of 10 Gy/5 fx) radiotherapy regimen. The patient has tolerated the radiotherapy and offered no complaints.

**Figure 3:**
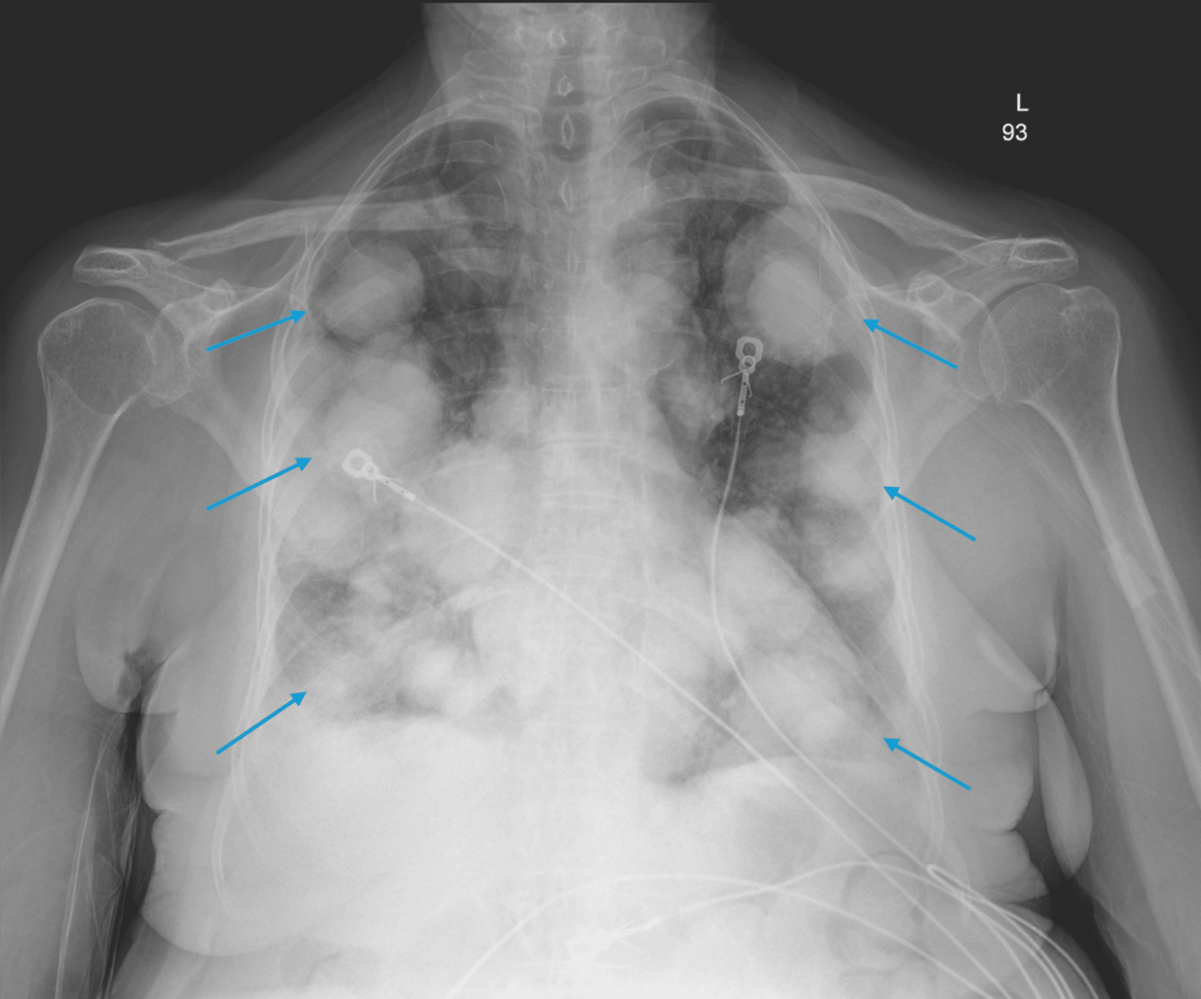
Chest X-ray on 4/20/2024 Mediastinum is midline. Normal cardiac silhouette. The blue arrow is pointing at numerous bilateral pulmonary masses. Bilateral costophrenic angles are blunted. No pneumothorax.

**Figure 4:**
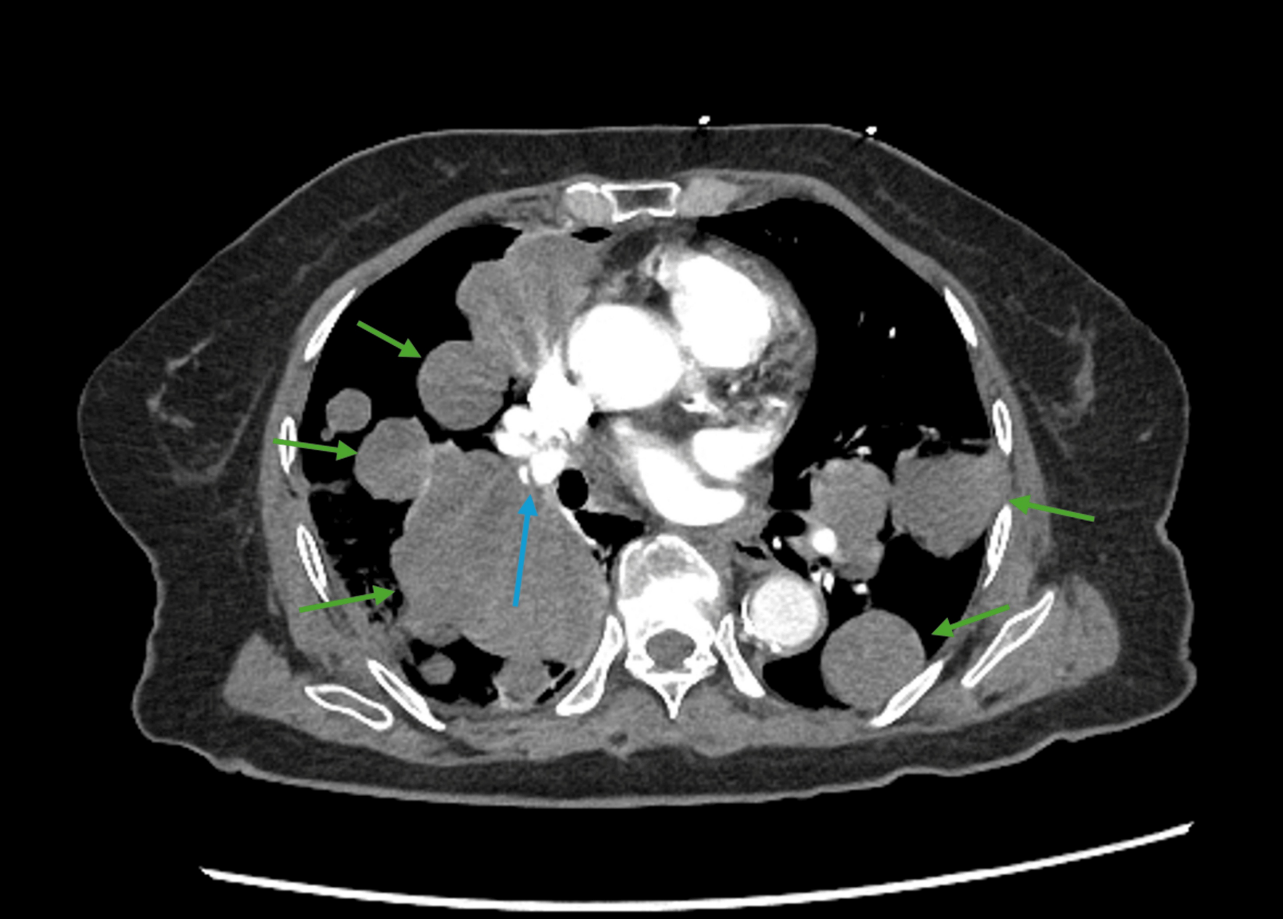
CT angiography of the chest with contrast The blue arrow points at a linear filling defect in the right lower lobe pulmonary artery consistent with acute pulmonary embolus. The green arrow points at multiple bilateral large soft tissue densities, indicating metastasis. The right lower lobe airspace is obscured by mass.

Upon presentation to the emergency department on 04/19/2024, a chest X-ray revealed numerous bilateral pulmonary masses, and a CT scan was consistent with metastatic malignancy given her history of sarcoma (Figures 3, 4). A CT angiography also confirmed an acute pulmonary embolism in the right lower lobe, despite the patient being on apixaban for anticoagulation. Considering leiomyosarcoma's tendency for hematogenous spread, the acute pulmonary embolism may be due to a solid tumor embolus rather than a typical clot embolus. As a result, anticoagulation might be of limited benefit in this situation.

An echocardiogram performed on 4/20/2024 revealed a normal-sized left ventricle with concentric remodeling and moderately reduced systolic function (EF 35-40%). The E/E' ratio of <10 suggested normal LVEDP. Significant wall motion abnormalities were noted, with severe hypokinesis in the apical septal and mid-anteroseptal walls, and moderate hypokinesis in the mid-inferoseptal wall.

The patient was admitted on 4/20/2024, with new diagnoses of heart failure with reduced ejection fraction (EF 35-40%) and pulmonary embolism. Her anticoagulation was switched from abixaban to enoxaparin sodium due to the failure of the former. An oncology consultation was requested to address the newly discovered metastatic leiomyosarcoma in the lungs.

Given the patient's advanced age, comorbidities, and the aggressive nature of her disease with pulmonary metastases, the multidisciplinary team determined that the patient was not a candidate for aggressive IV chemotherapy. The prognosis was deemed poor, with an estimated life expectancy of around 6 months. After careful consideration and discussion with the patient and her family, the decision was made to transition to palliative care. This approach aims to focus on symptom management and quality of life, rather than pursuing further aggressive treatments that the patient may not tolerate well.

The patient's current Karnofsky Performance Status is 70, indicating that she is able to care for herself but unable to carry on normal activities or do active work. This aligns with her palliative performance score of 60-70%. The Karnofsky score of 70 suggests that while the patient is still self-caring, she is experiencing significant impairment in her ability to perform normal daily activities or work tasks due to her illness. She has good social support and lives with her child, which is beneficial for her ongoing care.

This case highlights the challenges in managing aggressive malignancies in elderly patients with multiple comorbidities and emphasizes the importance of a multidisciplinary approach in determining the most appropriate care plan to maintain quality of life.

## Discussion

This case presents a complex scenario of an elderly patient with rapidly progressing leiomyosarcoma, highlighting several important aspects of cancer management in the geriatric population. The case demonstrates the unpredictable nature of leiomyosarcoma, even when initially graded as low-grade (FNCLCC grade 1). The rapid doubling of tumor size within two weeks of biopsy and the development of pulmonary metastases within five months of initial presentation make the unpredictable behavior of soft tissue sarcomas difficult to treat. This emphasizes the need for close monitoring and rapid intervention in such cases.

Perioperative radiotherapy is considered the gold standard for treating localized soft tissue sarcomas in the extremities, trunk, and head or neck regions [7]. Two randomized trials have shown improved local control rates when combining adjuvant radiotherapy with surgery for soft tissue sarcoma of the extremities and trunk [8,9]. Combining perioperative radiotherapy with chemotherapy remains controversial. Studies exploring this combination have shown promising results, despite increased toxicity. The Radiation Therapy Oncology Group (RTOG) 9154 trial demonstrated encouraging five-year survival rates with combined chemoradiotherapy, 56%, 64%, and 71%, respectively [10]. However, 97% of the subjects were noted to have grade 3 toxicities or greater. A study combining escalating doses of gemcitabine and ifosfamide with preoperative radiotherapy yielded impressive five-year outcomes for local control, distant metastasis-free survival, and overall survival rates above 80% [11]. However, despite these positive results, neoadjuvant chemoradiation for soft tissue sarcoma or leiomyosarcoma is still considered experimental and requires further investigation to establish its role in standard treatment protocols. As of today, there is no universally accepted first-line chemotherapy regimen for metastatic leiomyosarcoma. For patients with unresectable metastatic disease, the primary goals of treatment revolve around enhancing the quality of life.

The rapid progression of leiomyosarcoma from a localized tumor to metastatic disease within months poses a unique challenge. The patient's advanced age and multiple comorbidities, including hypertension, diabetes, and a history of pulmonary embolism, complicate treatment decisions. These factors, combined with her reduced functional status, limit the use of aggressive therapies like chemotherapy. The development of heart failure and a history of pulmonary embolism despite anticoagulation further compound the complexity of care. Balancing effective cancer treatment with quality-of-life considerations becomes crucial, especially given the poor prognosis.

## Conclusions

This case highlights a critical gap in current soft tissue sarcoma treatment protocols, particularly for elderly patients. While perioperative radiotherapy is standard for localized soft tissue sarcoma and adjuvant chemotherapy shows potential benefits in some studies, these approaches may not be suitable for all patients. The rapid progression of leiomyosarcoma in the left thigh with metastasis to the lung in this 84-year-old patient, coupled with her inability to tolerate chemotherapy due to cachexia and comorbidities, calls in the need for alternative treatment strategies. This scenario opens the door for developing more tailored protocols for patients who fall outside traditional chemotherapy criteria. Future research should focus on exploring less toxic, yet effective treatments, such as targeted therapies, immunotherapies, or modified radiotherapy regimens, which could offer better outcomes and quality of life for this vulnerable patient population. Such advancements could potentially bridge the treatment gap for patients who are currently left with limited options beyond palliative care.
